# Improved high-quality reference genome of red drum facilitates the processes of resistance-related gene exploration

**DOI:** 10.1038/s41597-023-02699-7

**Published:** 2023-11-07

**Authors:** Yongshuang Xiao, Jing Liu, Jiehong Wei, Zhizhong Xiao, Jun Li, Yuting Ma

**Affiliations:** Center for Ocean Mega-Science, Institute of Oceanology, Chinese Academy of Sciences, Qingdao, China

**Keywords:** Transcriptomics, Genome

## Abstract

*Sciaenops ocellatus* is among the most important artificially introduced farmed fish across 11 countries and regions. However, the frequent occurrence of extreme weather events and breeding escapes have placed great pressure on local marine biodiversity and ecosystems. We reported the *de novo* assembly and annotation with a contig N50 of 28.30 Mb using PacBio HiFi sequencing and Hi-C technologies, which resulted in a 283-fold increase in contig N50 length and improvement in continuity and quality in complex repetitive region for *S. ocellatus* compared to the previous version. In total, 257.36 Mb of repetitive sequences accounted for 35.48% of the genome, and 22,845 protein-coding genes associated with a BUSCO value of 98.32%, were identified by genome annotation. Moreover, 54 hub genes rapidly responding to hypoosmotic stress were identified by WGCNA. The high-quality chromosome-scale *S. ocellatus* genome and candidate resistance-related gene sets will not only provide a genomic basis for genetic improvement via molecular breeding, but will also lay an important foundation for investigating the molecular regulation of rapid responses to stress.

## Background & Summary

Red drum (*Sciaenops ocellatus*, Linnaeus, 1766, FishBase ID: 191), an estuarine fish species native to the western Atlantic Ocean from Massachusetts to northern Mexico in the United States, is one of the most important farmed fish species in the world^1–5^ (Figure S1). *S. ocellatus* is a temperate and saline fish with obvious characteristics such as a fast growth rate (≥10 °C, ~26 kg with a length of 1.23 metres according to wild angling in China), miscellaneous eating habits, a strong reproductive ability, high disease resistance, a high survival rate (2–33 °C), and low oxygen tolerance (≥2.2 mg/l), and can adapt to diverse habitats (the bay, intertidal, saline environments, and inland freshwater areas)^6–9^. The main breeding areas of *S. ocellatus* have expanded to 11 countries and regions covering the Atlantic, Pacific, Indian Ocean and Mediterranean regions through the globalization of breeding for more than 30 years, and the total breeding production (as of 2019) has reached 77008.58 tons with a product value of 1.96 × 10^8^ USD (www.fao.org/faostat) (Figure S1). The aquaculture of *S. ocellatus* has diversified modes mainly including seawater cage aquaculture, factory indoor aquaculture and nearshore pond aquaculture, of which seawater cage aquaculture accounts for more than 90% of the total aquaculture production^9–12^. In recent years, it was found that millions of *S. ocellatus* have escaped from seawater cages in the coastal areas of Zhejiang and Fujian, China, and individuals with mature gonads have been caught in natural marine areas^9,12^. Meanwhile, there have been increasing reports of *S. ocellatus* being caught in nonnative waters (off the coast of Mexico), with records in the Indo-West Pacific (*e.g*., Singapore and Thailand) and the Western Pacific (*e.g*., Taiwan Strait of China, East China Sea, South China Sea, and Korea)^13–17^. Wang *et al*.^17^ used an environmental DNA approach to detect a high abundance of *S. ocellatus* in the Jiaojiang Estuary and Sanmen Bay area in the East China Sea. Fishery resource surveys further revealed that alien *S. ocellatus* in the coastal waters of western Taiwan and the Indo-West Pacific have established breeding populations with significant invasive properites^9,15,16^. Therefore, it is necessary to obtain a high-quality chromosome-scale assembled genome and abundant resistance-related gene resources, which will facilitate studies of the molecular mechanisms of resistance in *S. ocellatus* and provide basic resources for assessment the subsequent rapid environmental adaptation and invasion genetics assessment of this species.

It is well known that high-quality reference genomes and complete annotations can provide important tools for population genomics and environmental adaptation genetics studies to efficiently mine genetic resources and accelerate the assessment of environmental adaptation^18–22^. Currently, only one version of the genome assembly for *S. ocellatus* has been published on the National Center for Biotechnology Information (NCBI) platform^23^. However, this version of the *S. ocellatus* genome was sequenced using the Illumina HiSeq. 2000 approach, which resulted in a heavily fragmented genome with a contig N50 length of only 99.71 kb. Therefore, an improved, high-quality version of the *S. ocellatus* genome is urgently required to support subsequent studies on the precise exploitation of genetic resources and genetic evaluation. PacBio SMRT sequencing employs a cyclic consensus long-line sequencing strategy to generate highly accurate HiFi reads, which can be combined with Hi-C assembly technology to enhance the continuity and quality of sequences, especially in complex repetitive regions of the *S. ocellatus* genome. This powerful combination of technologies has the potential to produce a more complete and accurate assembly of the *S. ocellatus* genome.

The integration of RNA sequencing technology and weighted gene coexpression network analysis (WGCNA) method enables a deeper understanding of the complex molecular mechanisms underlying various biological processes, including development, diseases, and environmental adaptation^20,24–27^. The combined analysis of RNA-seq and WGCNA has been successfully applied in the investigation of immune response, reproductive development, growth regulation, stress response mechanisms, and key gene identification in various fish species, such as *Siniperca chuatsi*^26^, *Sebastes schlegelii*^25^, *Cynoglossus semilaevis*^24^, *Lateolabrax maculatus*^27^, and *Paralichthys olivaceus*^20^. Some progress has been made in understanding the genetic and molecular regulatory mechanisms of the stress response of *S. ocellatus* under osmotic stress, such as high salt stress, and low salt stress. Although significant changes in expression associated with ion transport-related proteins upon exposure to osmotic stress have been detected for classic genes (*e.g. nka*, and *nkcc*), a more comprehensive analysis of gene modularity and connectivity between genes by gene coexpression network analysis has not been performed^5,28,29^. In this study, key modules and core genes were identified by analysing the relationship between *S. ocellatus* gene expression modules and low osmotic adaptation traits (32 psu, 16 psu, and 3 psu) through WGCNA, providing more effective data and a direction for supplementing and exploring the adaptation mechanism of *S. ocellatus* in adverse environments.

In this study, we used a combination of PacBio HiFi long-read sequencing and Hi-C technologies to assemble a high-quality chromosome-level *S. ocellatus* genome with a contig N50 size of 28.30 Mb. With this version of the high-quality genome assembly, we further improved the annotation of *S. ocellatus* repetitive sequences and protein-coding genes. In addition, based on the relevant RNA-seq datasets obtained from salinity stress gradient stress experiments, five key modules and 592 candidate genes responsive to hypoosmotic stress were identified by gene coexpression network analysis. The high-quality chromosome-scale assembly of the *S. ocellatus* genome and the identification of candidate stress-related gene sets not only provide a genomic basis for the genetic improvement of *S. ocellatus* via molecular breeding, but also lay an important foundation for studying the molecular regulation of the rapid stress response and the mechanism underlying rapid global environmental adaptation in *S. ocellatus*.

## Methods

### Sample collection, library construction and sequencing

**Fig. 1:**
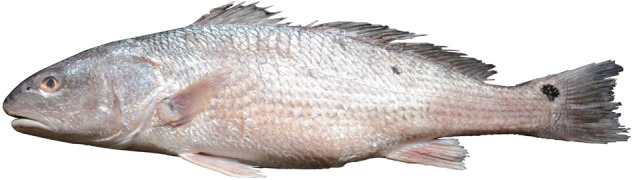
The image represented the adult *S. ocellatus* used for the genome sequencing and assembly process.

High-quality genomic DNA was extracted from fresh muscle tissue samples of a single female *S. ocellatus* from offshore Qingdao, Shandong Province, China (Fig. 1). The extracted DNA concentrations were assayed using a combination of Nanodrop (Thermo, NANODROP2000) and Qubit (Invitrogen, Qubit^TM^3Flurometer) methods, and then DNA integrity was examined using 1.5% agarose gel electrophoresis.

**Fig. 2:**
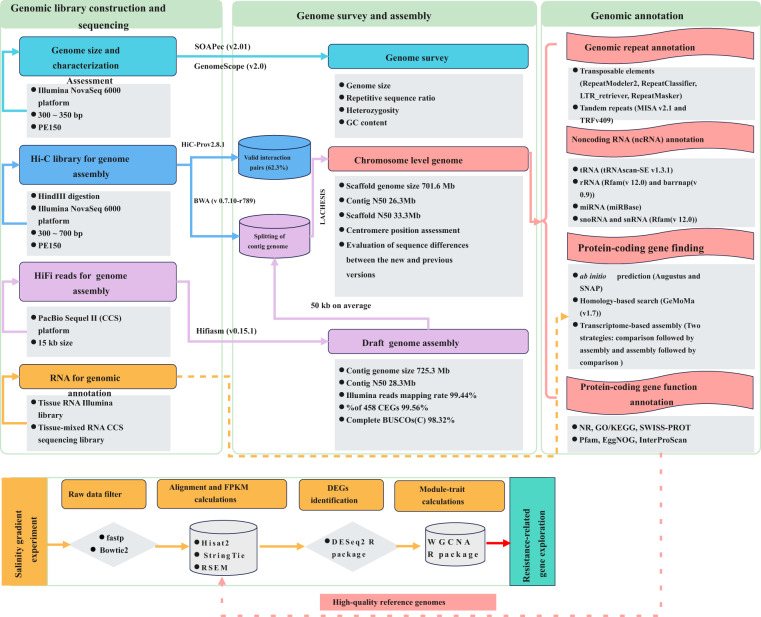
This diagram illustrates the workflow employed for the genome assembly and annotation of *S. ocellatus* in this study.

A short library (300–350 bp) was constructed using the NR604-VAHTS Universal V6 RNA-seq Library Prep Kit for Illumina (Vazyme, Illumina TruSeq DNA Library Prep Kit) and paired-end 150 bp (PE 150) sequenced using the standard protocol provided by the Illumina NovaSeq. 6000 platform (Illumina Inc., San Diego, CA, USA), yielding a total of 88.22 Gb of clear reads (90.7 Gb of raw reads) with a Q20 of 96.78% for assessing *S. ocellatus* genome size (Fig. 2, Table 1). High-molecular weight (HMW) gDNA was prepared into 15-kb libraries for PacBio HiFi read production using a standard protocol provided by PacBio (Pacific Biosciences, USA) (Fig. 2). The genomic libraries were sequenced on two cells using the self-testing high-precision CCS mode available as part of the PacBio Sequel II system. We obtained 54.78 Gb of HiFi long-reads with a read N50 length of 12.40 kb, resulting in 84-fold coverage of the *S. ocellatus* genome size (Fig. 2, Table 1). Hi-C libraries were generated using a process involving *HindIII* digestion of cross-linked high-quality DNA, 5′ biotin labelling and flat end repair to form chimeric junctions which were further physically sheared to a size into 300–700 bp fragments^30,31^. A total of 91.78 Gb (~135.31X) of paired-end clean reads were generated from the Hi-C library and sequenced using 150-bp paired-end sequencing on the Illumina NovaSeq. 6000 platform (Fig. 2, Table 1). Assisted genome annotation of tissue (muscle, gill, kidney, gonad, and liver) RNA Illumina libraries and mixed-tissue RNA CCS sequencing libraries yielded 39.73 Gb and 1.85 Gb of clean reads for genome annotation, respectively (Fig. 2, Table 1).

**Table 1:** Statistics for the sequencing data of the *S. ocellatus* genome.

Library type		Insert	Clean	Average	N50 Read	Sequencing
		Size (bp)	Data (Gb)	Read Length (bp)	Length (bp)	Coverage (X)
Genome assembly libraries	Illumina	350	88.22	150	150	130.06
	PacBio HiFi (CCS)	15,000	54.78	12,070	12,402	80.76
	Hi-C	300 ~ 700	91.78	150	150	135.31
	RNA-Seq (Illumina)	—	39.73	150	150	58.58
	RNA-Seq (CCS)	1000 ~ 6000	1.85	4,500	—	2.72
	Total	—	276.36	—	—	407.44

Note: Genome size estimated by genome survey (678.28 Mb) were used for sequencing coverage calculation.

*S. ocellatus* adults were collected from the aquaculture nets of Fuqiang Aquaculture Company (Shandong Province), China. Thirty-six individuals (1,025 ± 62 g) were transferred to the aquarium of the Institute of Oceanography, Chinese Academy of Sciences for temporary culture for 48 h. These fishes were allocated into nine tanks (400 L, 3 experimental groups × 3 replications) with water temperature, salinity, and light-dark cycle for 22 °C, 32 psu, and 13:11, respectively. After 48 h of temporary culture with aeration and satiety feeding, the fish were gradually exposed to three test experimental salinities (32, 16 and 3 psu) under 22 °C water, where 32 psu was set as the experimental control group. To elucidate the rapid response and *in vivo* regulation mechanisms in *S. ocellatus* under acute hypoosmotic stress, we sampled all control and experimental fish exposed to the set salinity in tanks and completed the experiment within 24 h. The fish were euthanized by complete immersion in an MS-222 bath (130 mg/L) followed by transection of the spine. Kidney and lamellae were removed from both branchial arches, which were immediately placed in 2 ml cryopreservation tubes and stored in liquid nitrogen for Illumina sequencing-based transcriptome experiments analysis. Gill and kidney tissues of *S. ocellatus* obtained from the three gradients of low-salt osmotic stress experiments (32 psu, 16 psu, and 3 psu) were subjected to library construction (300 ~ 500 bp) and sequencing (PE150) using standard protocols provided by the Illumina NovaSeq. 6000 platform. We obtained approximately 138.36 Gb of raw data including 916,304,804 reads for subsequent WGCNA (Fig. 2, Table 2).

**Table 2:** Statistics of sequencing data and data quality of the transcriptome of gill and kidney tissues from *S. ocellatus* under hypotonic stress.

Library type		Insert Size (bp)	Raw Data (bp)	Clean Data (bp)	Q20 (%)	Q30 (%)
RNA stress library (Illumina)	CKG32-1	300 ~ 500	6,961,559,946	6,899,732,360	98.25	95.15
	CKG32-2	300 ~ 500	7,908,393,366	7,841,908,254	98.25	95.15
	CKG32-3	300 ~ 500	7,934,439,960	7,865,641,259	98.26	95.16
	MSG16-1	300 ~ 500	7,311,457,750	7,249,751,821	98.23	95.10
	MSG16-2	300 ~ 500	8,445,570,430	8,373,120,678	98.26	95.17
	MSG16-3	300 ~ 500	8,022,673,790	7,951,343,065	98.22	95.09
	FWG3-1	300 ~ 500	7,842,575,184	7,774,237,865	98.13	94.89
	FWG3-2	300 ~ 500	8,008,243,928	7,940,906,641	98.19	95.00
	FWG3-3	300 ~ 500	7,155,048,024	7,097,511,856	98.27	95.21
	CKK-1	300 ~ 500	6,816,957,514	6,765,858,627	98.29	95.21
	CKK-2	300 ~ 500	7,435,136,112	7,378,664,375	98.31	95.24
	CKK-3	300 ~ 500	8,449,436,936	8,384,536,936	98.28	95.16
	MSK-1	300 ~ 500	8,320,239,222	8,256,728,630	98.29	95.22
	MSK-2	300 ~ 500	6,713,768,040	6,664,930,375	98.34	95.32
	MSK-3	300 ~ 500	7,383,948,320	7,326,191,578	98.34	95.32
	FWK-1	300 ~ 500	8,219,864,388	8,160,646,997	98.33	95.29
	FWK-2	300 ~ 500	7,990,297,578	7,931,148,971	98.31	95.28
	FWK-3	300 ~ 500	7,442,414,916	7,387,846,533	98.30	95.23
	Total	—	138,362,025,404	137,250,706,821	98.27	95.18

CKG, MSG, and FWG represented gill tissues at salinities of 32 psu, 16 psu, and 3 pus, respectively. CKK, MSK, and FWK represented kidney tissues at salinities of 32 psu, 16 psu, and 3 pus, respectively.

### Genome survey and assembly

**Fig. 3:**
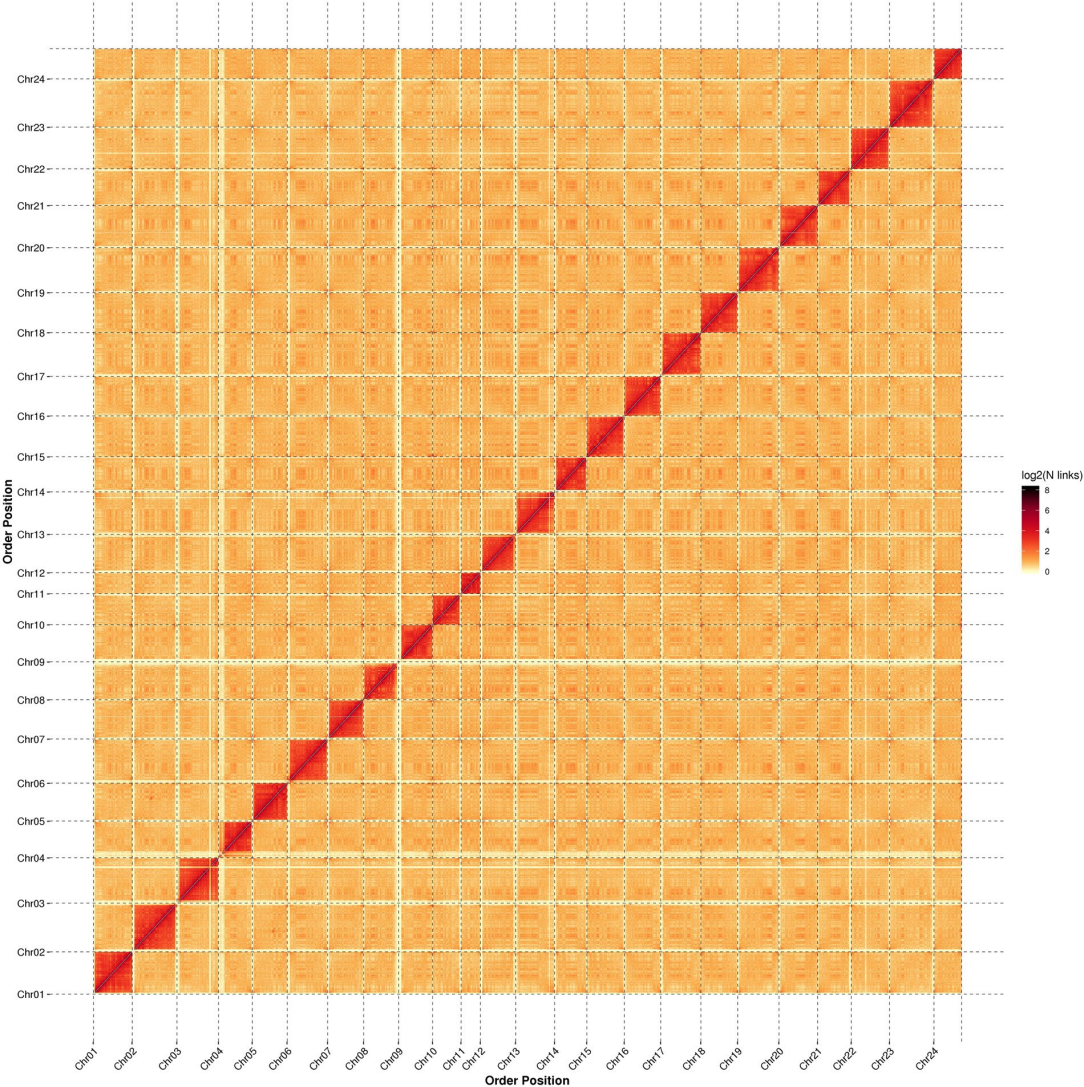
The heatmap displays the genome-wide Hi-C interactions in the assembly of the *S. ocellatus* genome.

A total of 88.22 Gb of Illumina clear reads was used to estimate the primary characteristics of the genome in SOAPec (version 2.01)^32^ and GenomeScope (version 2.0)^33^ software with 19 K-mer frequencies (Figure S2). The survey results showed that the genome size of *S. ocellatus* was 678.28 Mb with a repetitive sequence ratio, heterozygosity and GC content of 20.54%, 0.45% and 41.11%, respectively (Figure S3). Then, 54.78 Gb of HiFi long reads were used by the Hifiasm (v0.15.1)^34^ software with default parameters for assembly, and we obtained a genome size of 725.33 Mb comprising 316 contigs with a contig N50 length of 28.30 Mb for *S. ocellatus* (Table 3). The genome assembly was corrected by LACHESIS using 62.3% of uniquely mapped paired read pairs (259,072,033 pairs) from the Hi-C libraries, with scaffolds placed on chromosomes by clustering, sorting and orientation^35–37^ (Fig. 3). The highly contiguous genomes (contigs N50 26.29 Mb, scaffold N50 33.32 Mb) with a size of 701.63 Mb, accounting for 96.73% of the draft *S. ocellatus* genome, was assembled into the 24 corresponding chromosomes based on the karyotype analyses, which demonstrated the higher quality of this genome assembly compared to other previously published versions for further studying and understanding *S. ocellatus* species (Fig. 3, Table 3, Table 4).

**Table 3:** Comparative statistic of the *S. ocellatus* genome assembly with old ones.

Genome assembly	Evaluation Parameters	This study	Xu *et al*.
	Strategies	PacBio HiFi + Hi-C	Illumina + Hi-C
Contig	**Total Number**	316	49,517
	Total Length (Mb)	725.33	656.35
	**N50 (Mb)**	28.30	0.10
	N90 (Mb)	6.65	0.01
	Max Length (Mb)	34.55	0.89
Scaffold	Total Number	327	38,256
	Total Length (bp)	725.34	686.43
	**N50 (Mb)**	29.12	25.62
	N90 (Mb)	22.79	2.55
	Max Length (Mb)	35.48	32.50

**Table 4:** Statistics of 24 chromosomes of *S. ocellatus*.

Group	Number of Contigs	Cluster length (bp)	Order number	Order length (bp)
Chr01	5	31,213,420	3	31,019,771
Chr02	13	36,249,995	9	35,478,140
Chr03	7	33,636,801	5	33,107,011
Chr04	9	27,253,639	7	26,828,622
Chr05	7	28,081,432	6	27,850,847
Chr06	10	32,785,012	8	32,124,045
Chr07	11	28,685,924	11	28,685,924
Chr08	17	28,348,934	13	27,689,833
Chr09	13	27,909,217	10	27,085,710
Chr10	8	22,787,909	8	22,787,909
Chr11	11	16,120,791	5	15,492,323
Chr12	15	28,610,985	12	28,116,337
Chr13	19	30,981,467	19	30,981,467
Chr14	7	25,631,268	7	25,631,268
Chr15	8	30,125,623	6	29,804,343
Chr16	6	28,896,979	6	28,896,979
Chr17	9	32,389,980	7	31,838,488
Chr18	4	29,666,799	2	29,116,762
Chr19	7	32,745,365	7	32,745,365
Chr20	8	31,568,956	5	30,639,632
Chr21	6	27,173,879	4	26,875,209
Chr22	8	30,635,449	7	30,575,056
Chr23	15	38,064,778	6	35,187,052
Chr24	4	22,062,208	4	22,062,208
Total (Ratio %)	227 (47.29)	701,626,810 (96.73)	177 (77.97)	690,620,301 (98.43)

**Fig. 4:**
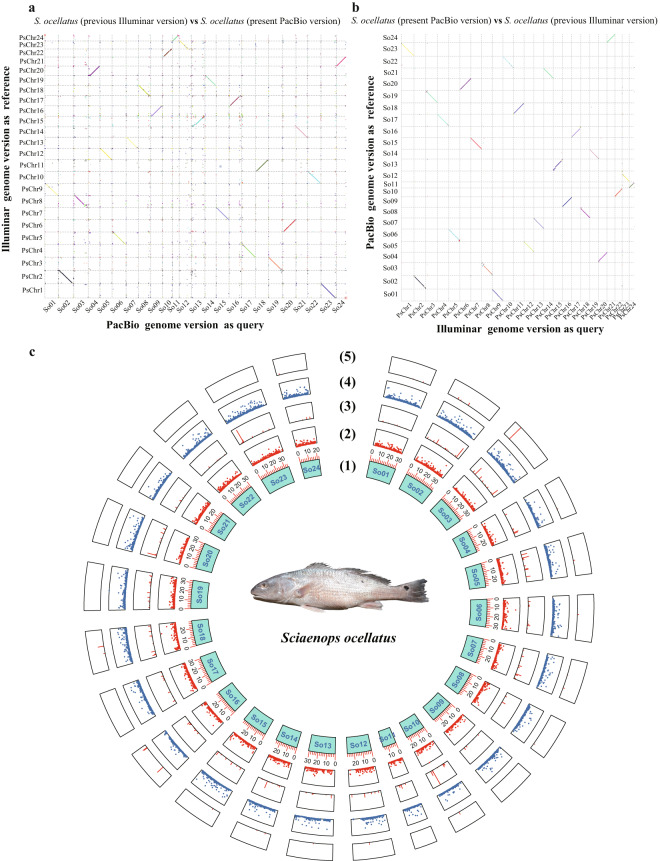
The covariance of sequence differences between the assembled PacBio genomes of *S. ocellatus* and the previously reported Illuminar version was shown on a genome-wide scale. (**a**) The reported *S. ocellatus* genome version vs the assembled version in the present study. (**b**) The assembled genome version of *S. ocellatus* vs the reported version. (**c**) Distribution of differential sites (assembled genome version of the present study as reference) on chromosomes with (1) Chromosome name, (2) Distribution of the number with deletions at the locus from 0 to 5000 bp, (3) Distribution of the number with deletions at the locus more than 5000 bp, (4) Distribution of the number with insertions at the locus from 0 to 5000 bp, (5) Distribution of the number with insertions at the locus more than 5000 bp.

The quality of the contig version of the *S. ocellatus* genome assembly was evaluated using CEGMA (v2.5)^38^ and BUSCO (v4.0, actinopterygii_odb10)^39^ assessments. The CEGMA assessment showed that 99.56% of the Core Eukaryotic Genes (CEGs) were present in the genome assembly. The BUSCO assessment indicated that 98.32% of the complete BUSCOs (Benchmarking Universal Single-Copy Orthologs) were present in the assembly (Table 5). TBtools software (v1.131, min_repeat_length 80 bp, max_repeat_length 350 bp, window_size 5000 bp, step_size 1000 bp) was used to confirm that the assembled *S. ocellatus* genome was composed of telomeric chromosomes, which were consistent with the previously reported chromosome C-banding results for *S. ocellatus*^40–42^ (Figure S4). Further statistics of genome-wide sequence difference covariance between the present genome version of *S. ocellatus* and the reported version using TBtools software (v1.131) showed that the assembled *S. ocellatus* genome was significantly superior to the previous version, displaying 202,685 deletion sites and 191,257 insertion sites^42^ (Fig. 4).

**Table 5:** Comparative assessment results of the *S. ocellatus* genome assembly (contig version) with previous version.

Assessment methods	Assessment parameters	This study	Xu *et al*.
Genome BUSCO	Complete BUSCOs	3,579 (98.32%)	95.9%
	Complete and single-copy BUSCOs	3,517 (96.62%)	91.8%
	Complete and duplicated BUSCOs	62 (1.70%)	4.1%
	Fragmented BUSCOs	6 (0.16%)	2.2%
	Missing BUSCOs	55 (1.51%)	1.9%
**Assessment methods**	**Assessment parameters**	**Quality value**	
BWA	Total clean reads number	303,805,182	
	Mapped clean reads	302,111,308	
	Clean reads mapping rate	99.44%	
	Properly mapped clean reads	297,063,212	
	Properly mapping rate	98.28%	
**Assessment methods**	**Assessment parameters**	**Quality value**	
CEGMA (v2.5)	Number of 458 CEG* present in assembly	456	
	Rate of CEGs present in assembly	99.56%	
	Number of 248 highly conserved CEGs present	247	
	Rate of 248 highly conserved CEGs present	99.60%

### Genomic repeat annotation

Transposable elements (TEs) were identified by a combination of homology-based and *de novo* approaches (Fig. 2). The RepeatModeler2 (v2.0.1)^43^ tool incorporating the RECON (v 1.08)^44^ and RepeatScout (v1.0.6)^45^ programs was first used to generate a customized *de novo* repeat library for the *S. ocellatus* genome. Then full-length long terminal repeat retrotransposons (fl-LTR-RTs) were identified using both LTRharvest (v1.5.9)^46^ (-minlenltr 100 -maxlenltr 40000 -mintsd 4 -maxtsd 6 -motif TGCA -motifmis 1 -similar 85 -vic 10 -seed 20 -seqids yes) and LTR_finder (v1.1)^47^ (-D 40000 -d 100 -L 9000 -l 50 -p 20 -C -M 0.9). The high-quality intact fl-LTR-RTs and nonredundant LTR library were then produced by LTR_retriever (v2.8)^48^. A nonredundant species-specific TE library was constructed by combining the *de novo* TE sequence library with the known Repbase (v19.06)^49^, REXdb (v3.0)^50^ and Dfam (v3.2)^51^ databases. Finally, RepeatMasker (v4.10)^52^ was employed to perform homology searches of nonredundant TE libraries to identify and classify TEs. Approximately 211,569,554 bp of TE sequences (375 full-length LTR sequences) accounting for 29.17% of the *S. ocellatus* genome were obtained. There represented an increase of 90.69 Mb compared to the previously reported version (Table 6)^23^. A total of 45.79 Mb of tandem repeats (6.31% of the whole genome) were also annotated by the Tandem Repeat Sequence Finder (v409, 1 1 2 80 5 200 2000 –d -h)^53^ and MIcroSAtellite Identification Tool (MISA v2.1)^54^ (Table 6).

**Table 6:** Comparative statistic of the *S. ocellatus* repeat sequences with previous version.

Class	This study				Xu *et al*
	Type	Number	Length (bp)	% in Genome	% in Genome
DNA	—	651,812	125,709,794	17.33	9.93
LTR	—	224,013	44,559,466	6.14	3.30
LINE	—	160,106	35,749,970	4.93	6.50
SINE	—	32,214	4,062,729	0.56	0.63
DIRS	—	9,566	1,487,595	0.21	—
	Total	1,077,711	211,569,554	29.17	17.61
Tandem Repeat	Microsatellite (1–9 bp units)	441,212	16,493,429	2.27	—
	Minisatellite (10–99 bp units)	8,381	9,670,097	1.33	
	Satellite (> = 100 bp units)	4,254	19,630,239	2.71	
	Total	453,847	45,793,765	6.31	

### Protein-coding gene identification and functional annotation

For noncoding RNA (ncRNA) annotation, tRNAs and rRNA were identified using tRNAscan-SE (v1.3.1)^55^ and barrnap (v0.9)^56^ based on the Rfam (v12.0)^57^ database, respectively. miRNA was identified using the miRBase database^58^. snoRNA and snRNA were predicted using Infernal (v1.1)^59^ based on the Rfam (v12.0)^57^ database. In total, 2,405 tRNAs, 2,986 rRNAs, and 582 miRNAs were predicted (Fig. 2, Table 7).

**Table 7:** Classification of ncRNAs of the *S. ocellatus* genome.

ncRNA type		Copy	Proportion in Genome
miRNA		582	0.0066%
tRNA		2405	0.0250%
rRNA	18S	556	0.1361%
	28S	580	0.3380%
	5.8S	540	0.0113%
	5S	1310	0.0201%
	Subtotal	2986	0.5371%
sRNA	ScaRNA	7	0.0002%
	HACA-box	58	0.0012%
	CD-box	102	0.0017%
	Splicing	422	0.0094%
	Subtotal	589	0.0125%

**Fig. 5:**
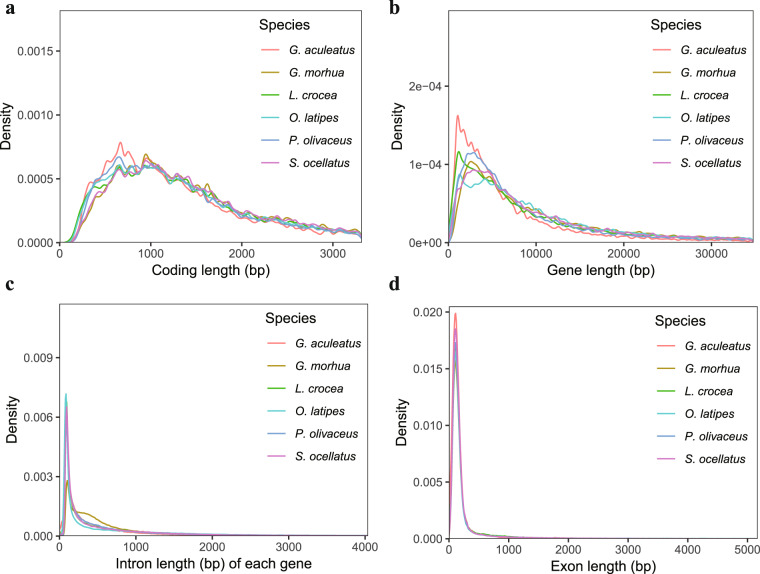
Comparison of genomic elements in closely related species. (**a**) Coding length distribution; (**b**) Gene length; (**c**) Intron length of each gene; (**d**) Exon length.

We used three approaches for gene prediction in the repeat-masked genome, including *ab initio* prediction, homology-based search, and transcriptome-based assembly (Fig. 2). Augustus (v2.4)^60^ and SNAP (2006-07-28)^61^ software were integrated for *ab initio* gene prediction and yielded 40,194 and 65,619 genes, respectively (Table 8). For the homologue-based approach, GeMoMa (v1.7)^62^ software was used with a reference gene model from the *Gasterosteus aculeatus*^63^, *Gadus morhua*^64^, *Larimichthys crocea*^65^, *Oryzias latipes*^66^, and *Paralichthys olivaceus*^67^ species, which annotated between 8,322 and 19,274 homologous genes (Table 8). Two strategies were applied for transcriptome-based gene annotation, which were comparison followed by assembly and assembly followed by comparison. We integrated Hisat (v2.0.4)^68^, StringTie (v1.2.3)^69^, and GeneMarkS-T (v5.1)^70^ software to map RNA sequence data to a reference genome and generate the assembly to predict 20,175 genes. PASA (v2.0.2)^71^ software was utilized to predict 20,510 genes using unigenes and full-length transcripts obtained by PacBio sequencing assembled with Trinity (v2.11)^72^. The gene models from these three approaches were merged using EVM software (v1.1.1)^73^ and updated by PASA, yielding a final total of 22,845 protein-coding genes with an average of 10.8 exons per gene in the *S. ocellatus* genome (Fig. 5, Table S1). The Venn diagram further showed that 19,629 genes (85.92%) were derived from transcriptome and homology-based predictions, indicating high quality of gene prediction for *S. ocellatus* (Figure S5). The BUSCO (v4.0)^39^ assessment showed that 97.9% of the complete BUSCOs were present in the assembly. This value indicated that the integrity of the assembled genome was high and significantly improved compared to the previously reported version (93.2%, complete BUSCOs) (Table 9). Meanwhile, the present version of the *S. ocellatus* genome (22,845) included 2,792 more annotated protein-coding genes than the previous version (20,053) (Table 10).

**Table 8:** General statistics of the predicted protein-coding genes for *S. ocellatus*.

Gene prediction methods	Prediction software	Species	Gene number
*Ab initio*	Augustus	—	40,194
	SNAP	—	65,619
Homology-based	GeMoMa	*G. aculeatus*	8,322
		*G. morhua*	18,742
		*L. crocea*	15,337
		*O. latipes*	15,858
		*P. olivaceus*	19,274
RNAseq	GeneMarkS-T	—	20,175
	PASA	—	20,510
Integration	EVM	—	22,845

**Table 9:** Comparative assessment results of the *S. ocellatus* gene annotation (scaffold version) with previous version.

Assessment methods	Assessment parameters	This study	Xu *et al*.
Genome BUSCO	Complete BUSCOs	3,284 (97.91%)	93.2%
	Complete and single-copy BUSCOs	3,212 (95.77%)	88.3%
	Complete and duplicated BUSCOs	72 (2.15%)	4.9%
	Fragmented BUSCOs	18 (0.54%)	2.7%
	Missing BUSCOs	52 (1.55%)	4.1%
**Assessment methods**	**Assessment parameters**	**Number**	**% in the gene annotation**
Hisat2	Clean data of RNA-seq covering the exon region	213,361,548	83.03
	Clean data of RNA-seq covering the intron region	16,753,943	6.52
	Clean data of RNA-seq covering the intergenic region	26,844,444	10.45
	Total clean reads of RNA-seq	256,959,935	100.00

**Table 10:** Comparative statistic of the *S. ocellatus* functional annotation with previous version.

Database	Number	Percentage (%)	Number	Percentage (%)
	(this study)	(this study)	(Xu *et al*.)	(Xu *et al*.)
Total	22,845	100.00	20,053	100.00
GO	20,491	89.70	15,030	74.95
KEGG	20,821	91.14	17,866	89.09
Swissprot	21,750	95.21	19,597	97.73
TrEMBL	22,802	99.81	20,037	99.92
InterPro	—	—	19,055	97.73
KOG	15,974	69.92	—	—
Pfam	21,690	94.94	—	—
eggNOG	20,725	90.72	—	—
NR	22,807	99.83	—	—
Unannotated	33	0.15	14	0.07

**Fig. 6:**
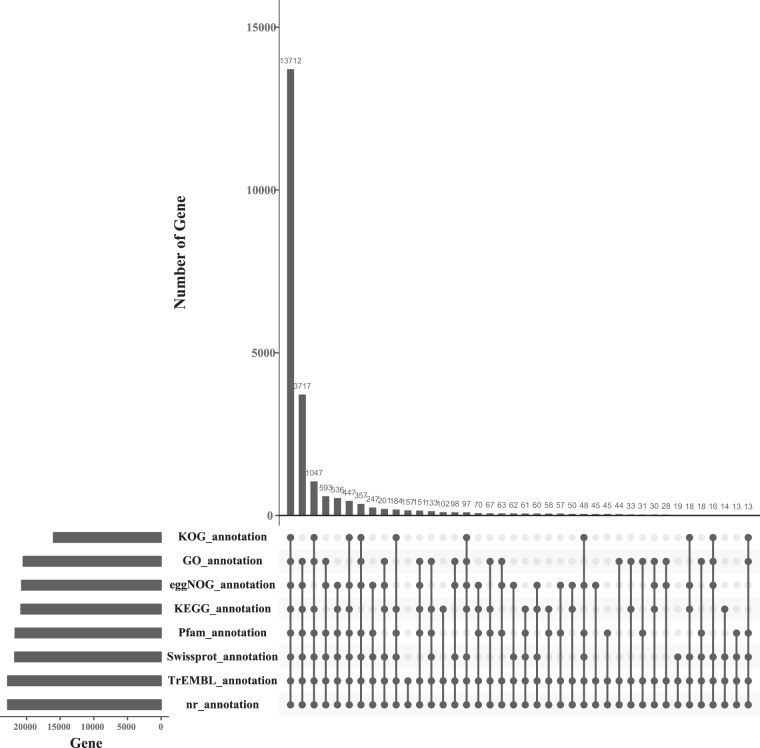
Upset plot showing the functional annotation of genes in the assembled genomes of *S. ocellatus* based on different databases.

The protein-coding genes were functionally annotated by aligning the obtained protein-coding genes with the NCBI Nonredundant protein (NR) (202009, ftp://ftp.ncbi.nlm.nih.gov/blast/db), GO (20200615, http://geneontology.org)^74^, KEGG (20191220, http://www.genome.jp/kegg)^75^, SWISS-PROT (202005, http://ftp.ebi.ac.uk/pub/databases/swissprot)^76^, and Pfam (v33.1, http://pfam.xfam.org)^77^ databases using Blastp (v2.2.26)^78^ and Diamond (v0.8.22)^79^ software with an e-value threshold of 1e-5, which yielded the percentage of genes with functional annotations ranging from 69.92% to 99.83% (Table 10). Then, according to the above annotation results combined with the EggNOG (v5.0, http://eggnog5.embl.de/download/eggnog_5.0/) database for further verification and additional annotation of protein-coding genes, a total of 22,812 protein coding genes were functionally annotated (99.86% of the total number of predicted genes) (Table 10). A total of 13,712 of these annotated protein-coding genes received common functional annotations in the eight functional datasets described above (Fig. 6). Protein structural domains and motifs were also annotated using the InterProScan (v5.34-73.0)^80^ database, yielding 59,941 structural domains and 2,077 motifs, respectively.

### Weighted gene coexpression network (WGCA) construction

For high-throughput transcriptome sequencing to obtain raw data, we performed the following standard processing protocol. First, we utilized fastp (v0.18.0)^81^ and Bowtie2 (v 2.2.8)^82^ software to filter low-quality and ribosomal RNA (rRNA) data from raw data (138.36 Gb, 916,304,804 reads), respectively. The paired-end clean data (137.25 Gb, 908,288,508 reads) (Q30 ≥ 95.2%) were aligned to the *S. ocellatus* genome using the Hisat2 (v2.0.5)^68,83^ software with default parameters, achieving an alignment rate of 95.19% (864,561,166 reads). Then, StringTie (v1.3.1)^69^ program was employed to determine the count of reads aligned to each gene in the reference genome. Subsequently, fragment per kilobase of transcript per million mapped reads (FPKM) values were calculated using RSEM^84^ software to estimate gene expression levels. Differential expression analysis between two groups was conducted using the DESeq. 2 R package^85^ (Fig. 2). According to the expression criterion (|*log2fold change*| ≥ 1) and a *p-adj value* < 0.05, we detected a total of 334, 59, 329, 527, 30, and 334 differentially expressed genes (DEGs) in the comparisons of FWG3 vs. CKG32, MSG16 vs. CKG32, FWG3 vs. MSG16, FWK vs. CKK, MSK vs. CKK, and FWK vs. MSK, respectively (Table 2) (Supplementary DEGs_Set.xlsx).

**Fig. 7:**
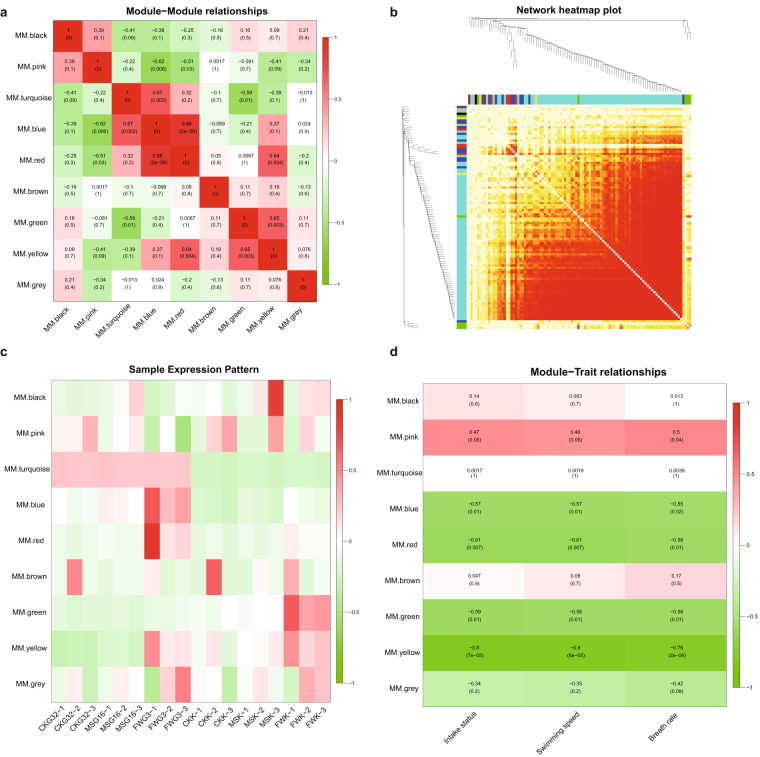
WGCNA analysis of hypoosmotic stress experiments using RNA high-throughput sequencing. (**a**) It represented module-to-module relationships, with stronger colours representing significant correlations. (**b**) Constructed gene co-expression clustering tree and co-expression topology heat map. Note, stronger colours represented higher expression similarity between genes. (**c**) It referred to the expression pattern samples, which represented the expression trend of the module eigengenes in different samples, and the similarity of the colours represented the consistent expression trend of the sample genes. (**d**) The constructed relationship matrix of modules-traits, where positive values represented module eigengenes positively correlated with the trait and negative values represented their negative correlation with the trait. Note, significance was taken at 0.05.

To identify the components of gene modules in response to hypo-osmotic stress for *S. ocellatus*, we constructed correlations between gene expression modules and hypo-osmotic acclimation traits (intake status, swimming speed and respiratory rate) using the WGCNA method based on the RNA-seq dataset obtained from the salinity stress gradient experiment (32 psu, 16 psu, and 3 psu). The WGCNA (v1.47)^86^ package in R was used to construct a coexpression network. After filtering out 7,946 genes, 16,034 gene expression values were utilized in WGCNA to create coexpression modules (Figure S6). The automatic network construction function blockwiseModules (power = 7, TOMType = unsigned, mergeCutHeight = 0.1, minModuleSize = 50) was employed with the default settings for the remaining parameters. This resulted in the clustering of genes into 9 related modules, where genes within the same module were considered to exhibit similar expression patterns (Figure S7). We used Pearson’s method to further statistically compute the correlation coefficients for module-to-module correlations and gene-to-module eigenvalue correlations, and utilized Student’s t test to obtain *p* values. Four significantly correlated module pairs were detected for turquoise-blue (*r = *0.67, *p* < 0.001), red-blue (*r* = 0.88, *p < *0.001), green-yellow (*r = *0.65, *p < *0.005), and yellow-red (*r* = 0.64, *p* < 0.005) (Fig. 7). Five modules significantly correlated with traits (positive correlation: pink module *r* = 0.47 ~ 0.50; negative correlation: blue module *r* = −0.55 to −0.57; red module *r* = −0.58 ~ −0.61; green module *r* = −0.58 ~ −0.59, and yellow module *r* = −0.76 ~ −0.8) were identified based on Spearman correlation coefficients between module eigenvalues and traits (Fig. 7).

### Module functional enrichment and identification of key hub genes

To further identify the core module (pink, blue, red, green, and yellow) genes closely associated with hypoosmotic stress traits, we integrated the gene connectivity results within the module (All.kWithin) and used the CentiScape^87^ plugin in Cytoscape (v3.10.0)^88^ for core gene selection using the closeness, degree, and betweenness methods. We obtained 41, 168, 105, 150 and 128 core genes for the pink, blue, red, green and yellow modules, respectively, and annotated the biological processes and KEGG pathways of candidate genes in Metascape^89^ (min_overlap 1, *p-value* cutoff 0.05, min_enrichment 1.5) (https://metascape.org/) (Supplementary hub_gene_set.xlsx, Figure S8).

GO biological process enrichment of the core genes in the pink module with a positive module–trait correlation indicated that *S. ocellatus* rapidly initiated protein translation processes, including cytoplasmic translation, biosynthesis of the ribonucleoprotein complex, and the haemoglobin biosynthetic process in response to hypoosmotic environmental stress (Figure S8). KEGG functional enrichment analyses further showed that hypotonic stress induced ribosomal complex assembly and protein translation processes.

GO biological process enrichment results of the core genes in the blue module with a negative module–trait correlation indicated that hypotonic stress initiated biological processes such as cellular response to stress, aerobic respiration, stress granule assembly, apoptotic signaling pathway, and the acetyl-CoA biosynthetic process from pyruvate, in addition to initiating the protein translation process. In addition, KEGG enrichment analysis also confirmed that hypotonic induction initiated pathways such as NOD-like receptor signalling pathways, oxidative phosphorylation, and protein processing in the endoplasmic reticulum (Figure S8).

GO enrichment analysis of the red module also showed that hypotonic stress promoted biological processes such as the ncRNA metabolic process, the biosynthetic process of nucleobase-containing compounds, negative regulation of the MAPK cascade, regulation of cellular response to stress, and response to corticotropin-releasing hormone, in addition to inducing protein translation processes (Figure S9). The KEGG enrichment results further showed that hypotonic stress induced the MAPK signalling pathway, as well as energy metabolism (such as the citrate cycle-TCA cycle) and stress response (protein processing in the endoplasmic reticulum), causing *S. ocellatus* to have a hypotonic response with a low intake rate, and low respiration rate and so on.

The GO biological process enrichment results for the green module showed that hypotonic stress initiated the cellular mitochondrial structure and electron respiratory chain complex assembly process while promoting the protein translation process, which might be compatible with a decline in salt ion efflux function *in vivo* and reduction in endogenous energy demand under hypotonic conditions. The KEGG enrichment results confirmed that hypotonicity promotes the citric acid cycle and amino acid metabolism reactions in addition to inducing organismal ribosome assembly as well as oxidative phosphorylation reactions (Figure S10).

Both the GO biological process enrichment and KEGG enrichment results for the yellow module, which was significantly correlated with the red and green modules, confirmed that hypotonic induction promoted ribosome small- and large-subunit biogenesis as well as protein translation, and accelerated DNA-guided transcription and nucleocytoplasmic translocation biological processes (Figure S11).

**Fig. 8:**
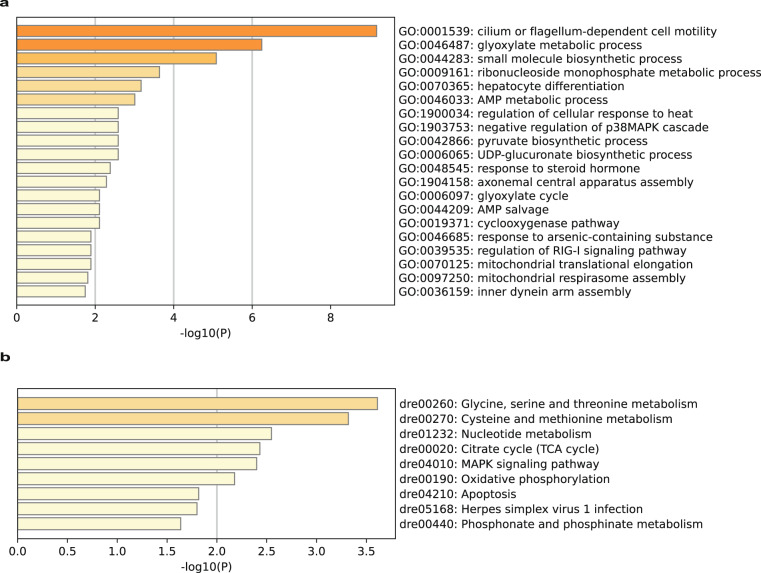
The biological processes GO enrichment and KEGG enrichment of differentially expressed hub genes. (**a**) GO enrichment for biological processes of 54 hub genes. (**b**) KEGG enrichment of 54 hub genes.

We intersected the core set of genes in the modules closely associated with the traits obtained in the above analyses with the set of DEGs obtained from the hypotonic induction experiments, and finally obtained a set of 54 key hub genes associated with hypotonic stress tolerance in *S. ocellatus*. GO enrichment of core DEGs revealed that hypotonic stress promoted cell-autonomous motility and environmental signalling processes, inducing multiplexed metabolic processes (such as the glyoxylate metabolic process, small molecule biosynthetic process, and ribonucleoside monophosphate metabolic process) (Fig. 8). The KEGG enrichment results further revealed that hypotonic stress stimulated the glycine, serine and methionine metabolic antiaging pathways^90^, as well as the endogenous taurine-synthesized cysteine and methionine metabolic pathways^90,91^, which in turn contributed to the stress tolerance and environmental adaptation of *S. ocellatus*.

## Data Records

The sequencing datasets and genome assembly have been deposited into the public database. Genome sequencing data (Illumina, PacBio, Hi-C and RNA-seq data) and transcriptomic sequencing data used for Genome assembly has been deposited in the SRA at NCBI SRP465111^92^.

The transcriptomic sequencing data for WGCA has been deposited in the SRA at NCBI SRP465080^93^.

The final chromosome assembly and genome annotation files and associated supplementary results have been deposited in the GCA at Genbank GCA_033000465.1^94^ and Figshare^95^.

## Technical Validation

The Merqury (v1.3)^96^ software was used to assess the quality of the genome, and the consensus quality value (QV) and completeness statistic values with 53.38 and 93.23% indicated that the assembled genome possessed a high level of accuracy and completeness (Figure S12). The Illumina sequencing reads were also aligned to the assembled genome using BWA (v0.7.8)^97^. The results showed that 99.44% of the reads were successfully mapped to the assembly, and 98.28% of the reads were properly mapped, indicating a high mapping efficiency (Table 5). The RNA-seq clean data was further aligned to the assembled genome using Hisat2 (v2.0.5)^68^. The results showed that 83.03% of the transcriptome data mapped to the predicted exons, demonstrating the high accuracy of the prediction model used (Table 9).

### Supplementary information

Supplementary DEGs_Set

Supplementary hub_gene_set

Supplementary Information

## Data Availability

All commands and pipelines used in data processing were executed according to the manual and protocols of the corresponding bioinformatics software.
