# Supplementary material for: Improved high-quality reference genome of red drum facilitates the processes of resistance-related gene exploration
Source: Sci Data. 2023 Nov 7;10:774. doi: 10.1038/s41597-023-02699-7 (PMC10630468; doi:10.1038/s41597-023-02699-7)
Supplement: Supplementary file 3 — Supplementary Information [file 41597_2023_2699_MOESM3_ESM.pdf]

Supplementary material

Improved high-quality reference genome of red drum facilitates the processes of resistance-related gene exploration

Yongshuang Xiao[1], Jing Liu[1*], Jiehong Wei[1], Zhizhong Xiao[1], and Jun Li[1*], Yuting Ma[1]

[1]Center for Ocean Mega-Science, Institute of Oceanology, Chinese Academy of Sciences, Qingdao, China

∗Correspondence:
jliu@qdio.ac.cn (J.L.) , and junli@qdio.ac.cn (J.L.)

Supplementary Table number: 1

Supplementary Figure numbers: 12

Species	Number	Average gene length (bp)	Average exon length (bp)	Average CDS length (bp)	Average intron length (bp)	Average exon per gene
S. ocellatus	22,845	17,018.04	2,789.08	1,854.48	14,228.96	10.84
L. crocea	22,853	16,246.1	2,338.62	1,704.26	13,907.48	10.13
G. morhua	23,303	16,560.21	3,182.16	1,824.49	13,378.05	11.31
P. olivaceus	22,927	14,821.1	2,727.17	1,653.18	12,093.94	10.37
G. aculeatus	20,705	92,44.33	1,713.17	1,548.32	7,531.16	10.65
O. latipes	22,063	17,982.51	2,888.79	1,638.63	15,093.72	9.49

Table S1. General statistics of the genomic characterization between *S. ocellatus* and the other closely related species' genomes.

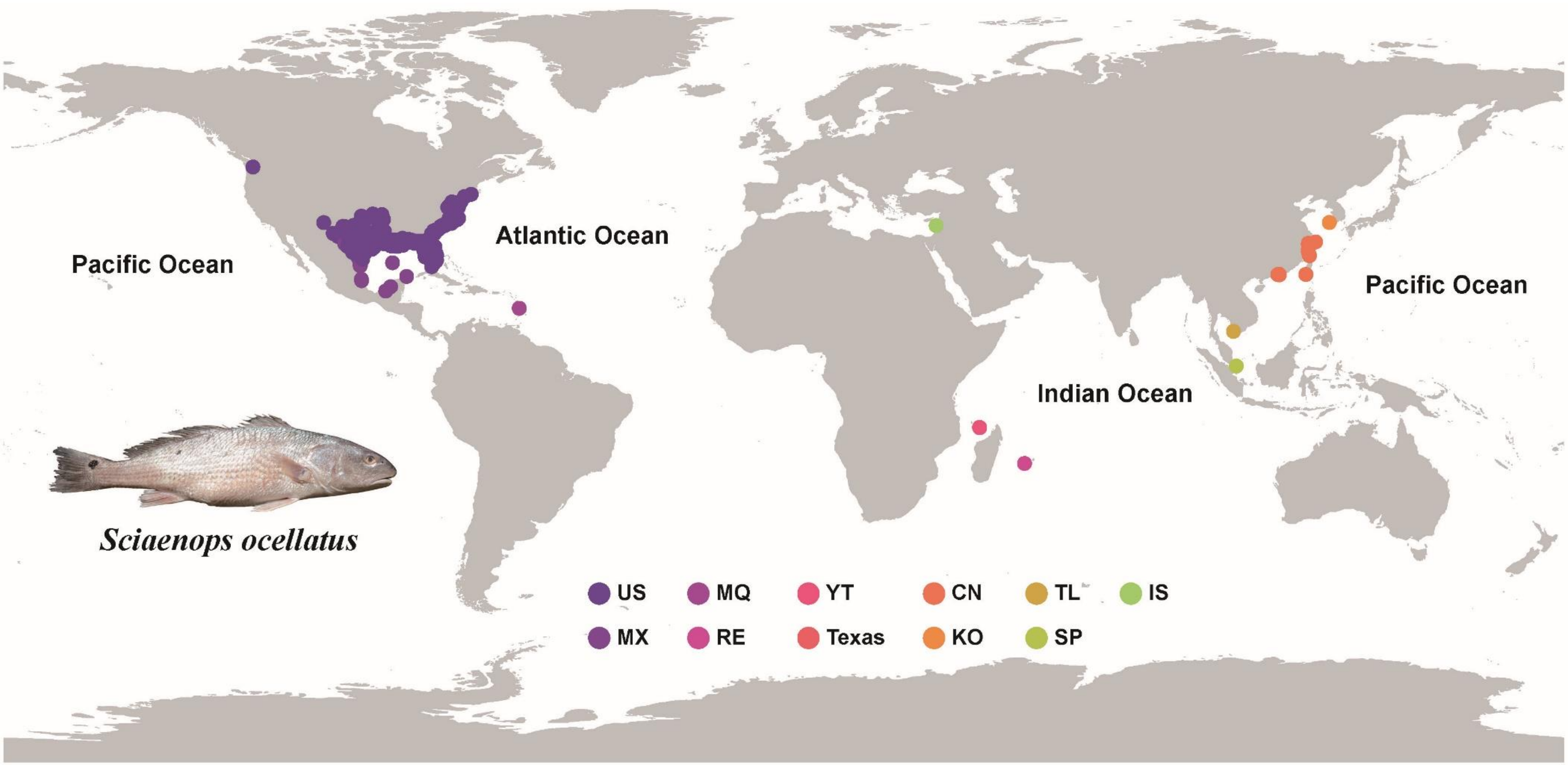

Figure S1. Distribution map of worldwide breeding and field catch records of *S. ocellatus*. US: United States of America, MX: Mexico, MQ: Martinique, RE: La Réunion, YT: Mayotte, CN: China, KO: Korea, TL: Thailand, SP: Singaporean, IS: Israeli.

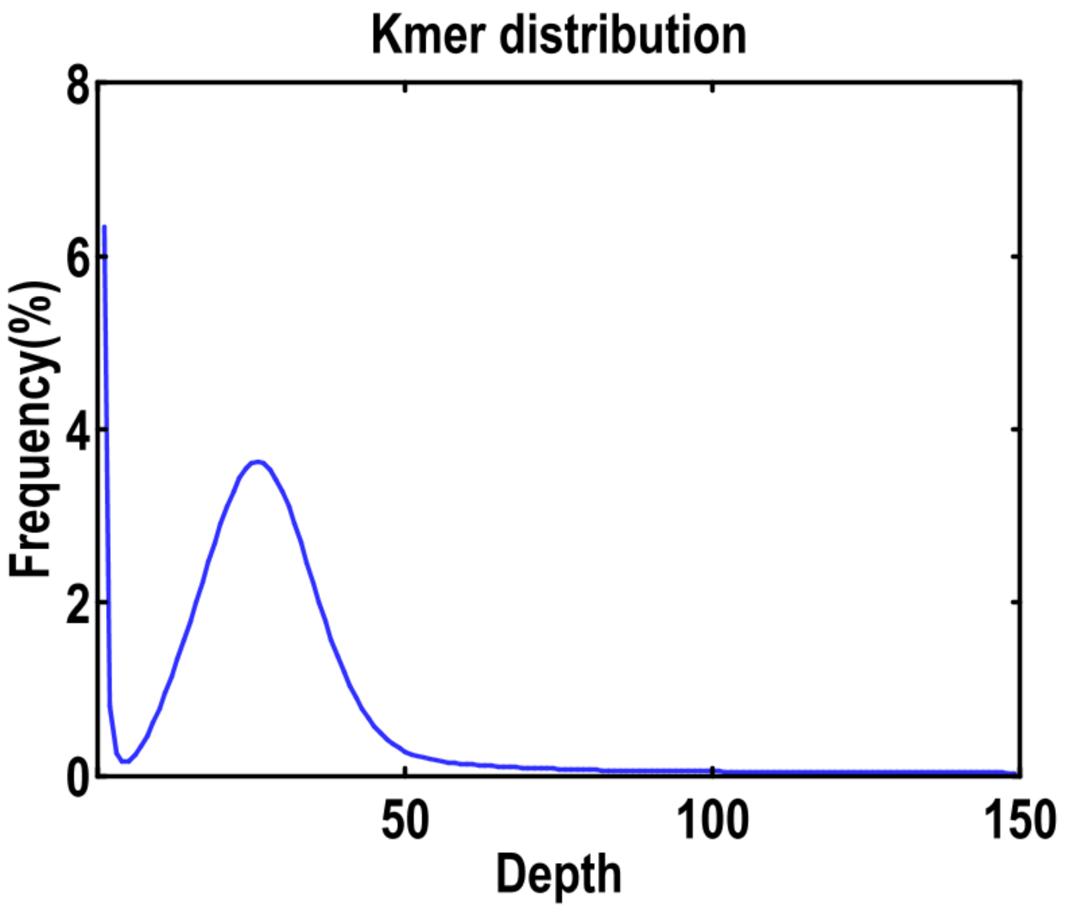

Figure S2. Frequency distribution of 19-mers in the *S. ocellatus* genome.

The X-axis was the k-mer depth, and Y-axis represented the frequency of the k-mer for a given depth.

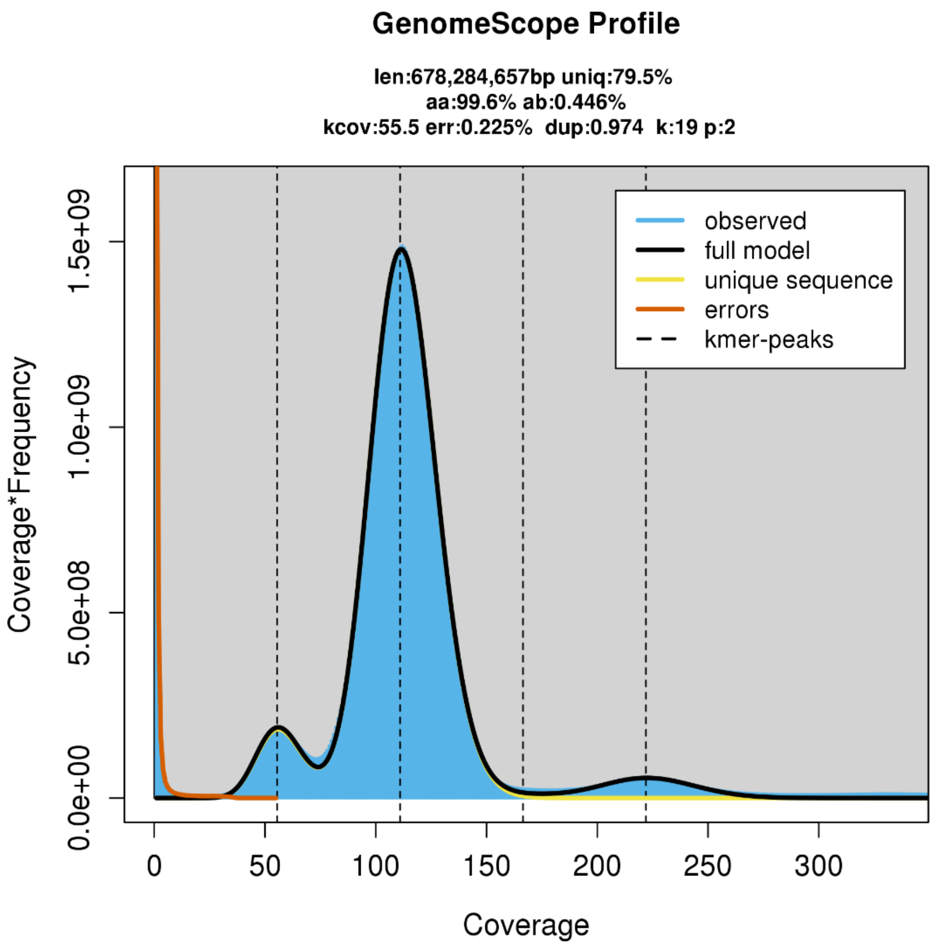

Figure S3. Genomic survey results and parameters for the *S. ocellatus* genome.

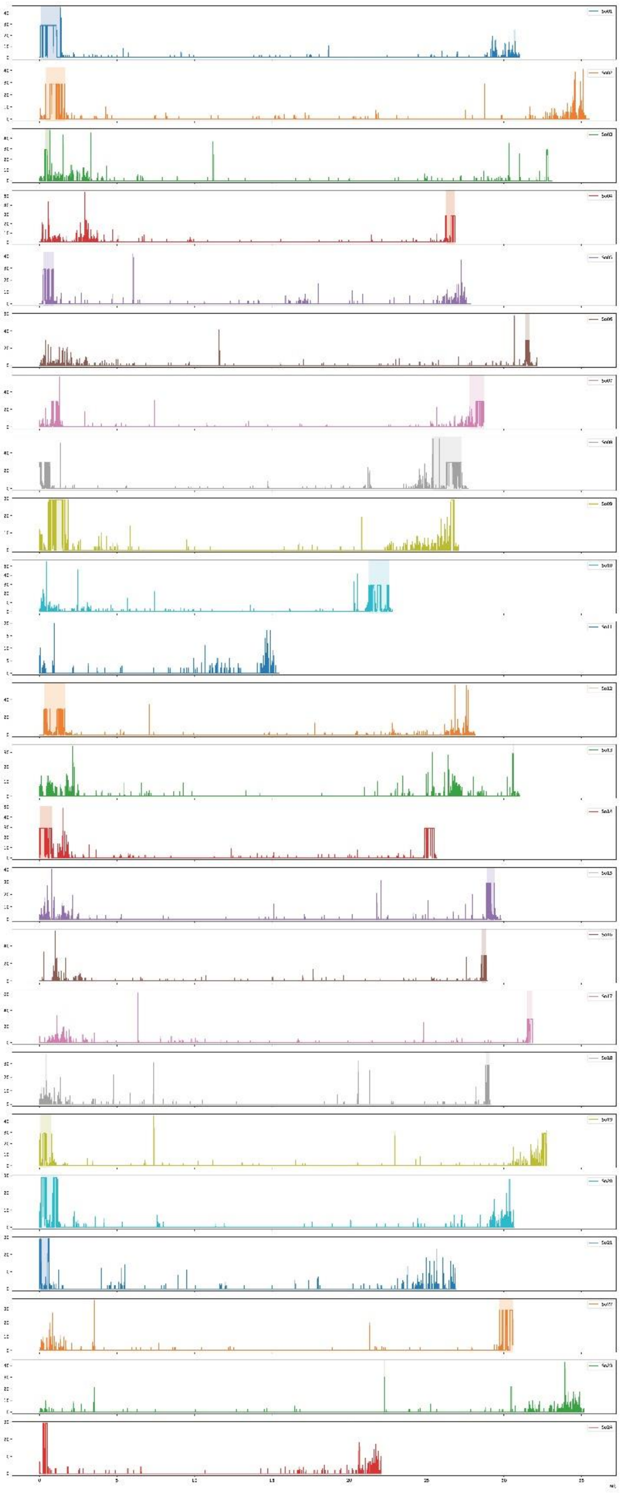

Figure S4. Identification and characterization of telomeric chromosomes in the assembly of the *S. ocellatus* genome.

The X-axis represented the length and position of the chromosome, the Y-axis represents the frequency of telomeric characteristic sequences.

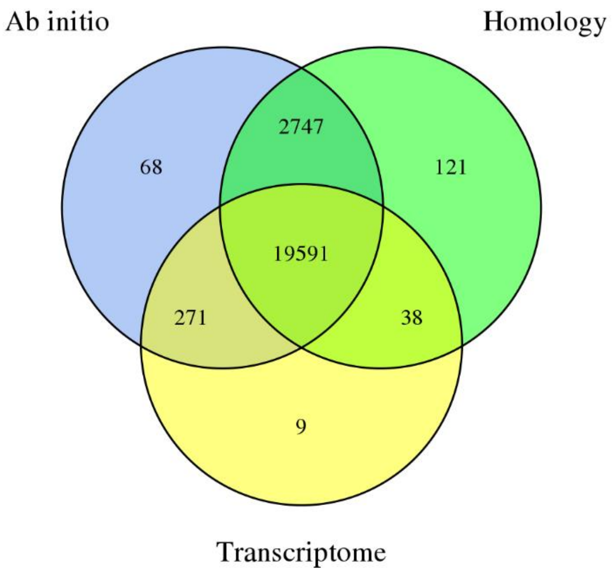

Figure S5. Comparative analysis of three gene prediction methods: ab initio prediction, homology-based search, and transcriptome-based assembly.

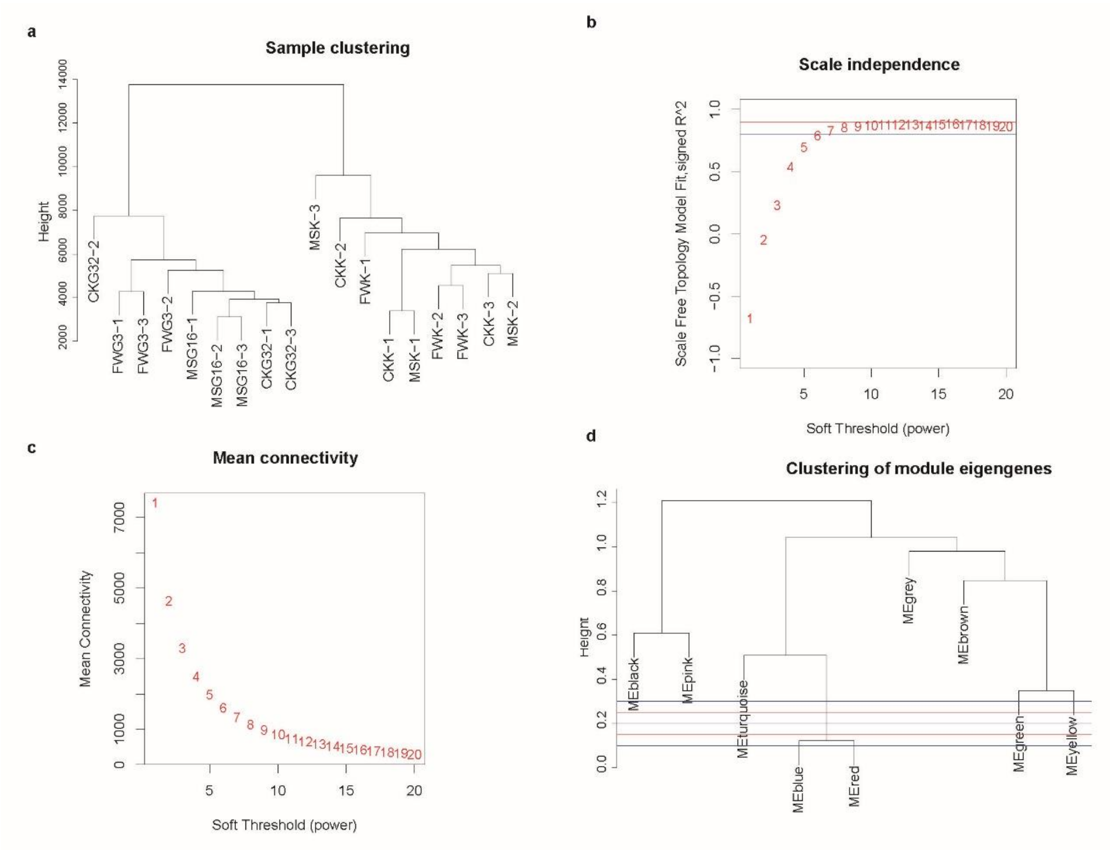

Figure S6. Scale-free topological analysis to determine optimal beta and co-expression matrix and module determination for WGCNA. (a) Sample clustering, (b) Soft thresholding powers determination (R^2> 0.8), (c) Mean connectivity determination, (d) Clustering of module eigengenes based on similarity of expression between genes.

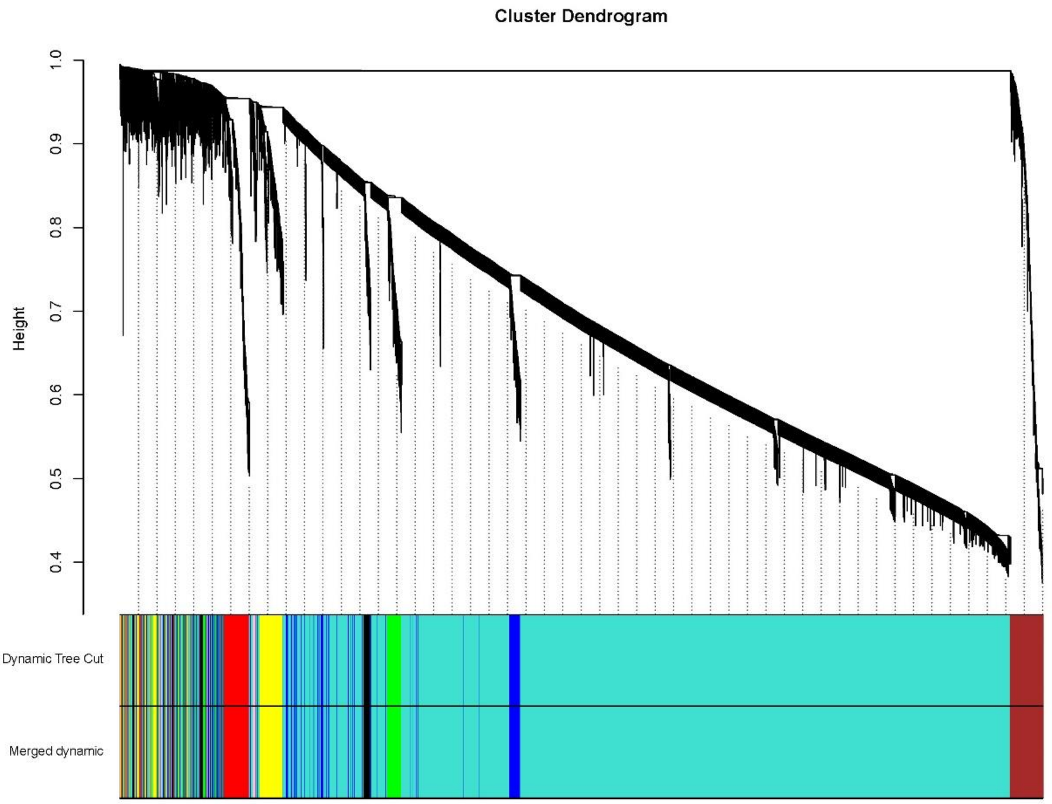

Figure S7. Construction of gene dendrograms and module colors for identifying co-expression modules in WGCNA.

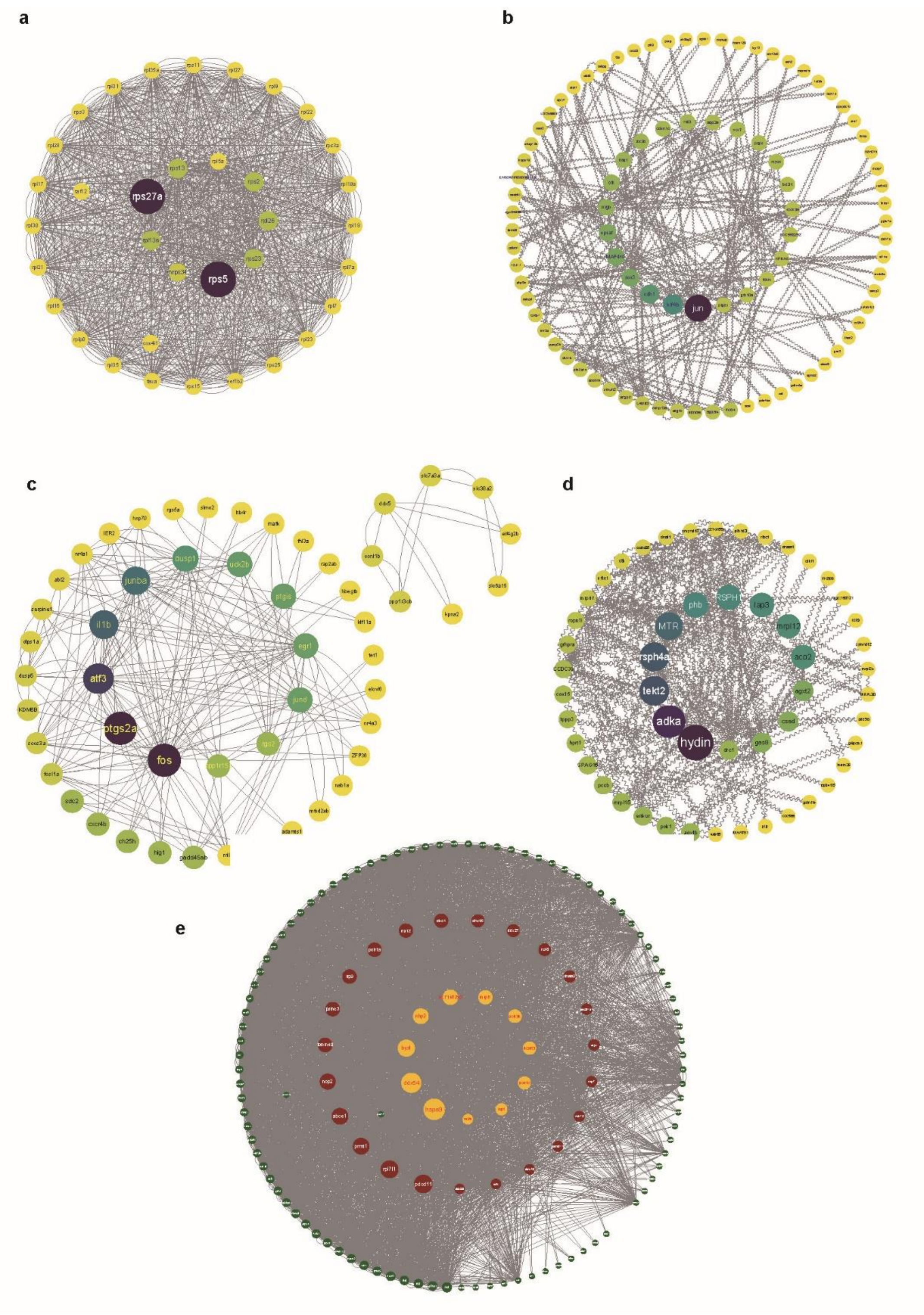

Figure S8. Core gene selection from modules using Closeness, Degree, and Betweenness methods.

(a) Core genes arrangement for pink module, (b) Core genes arrangement for blue module, (c) Core genes arrangement for red module, (d) Core genes arrangement for green module, (e) Core genes arrangement for yellow module.

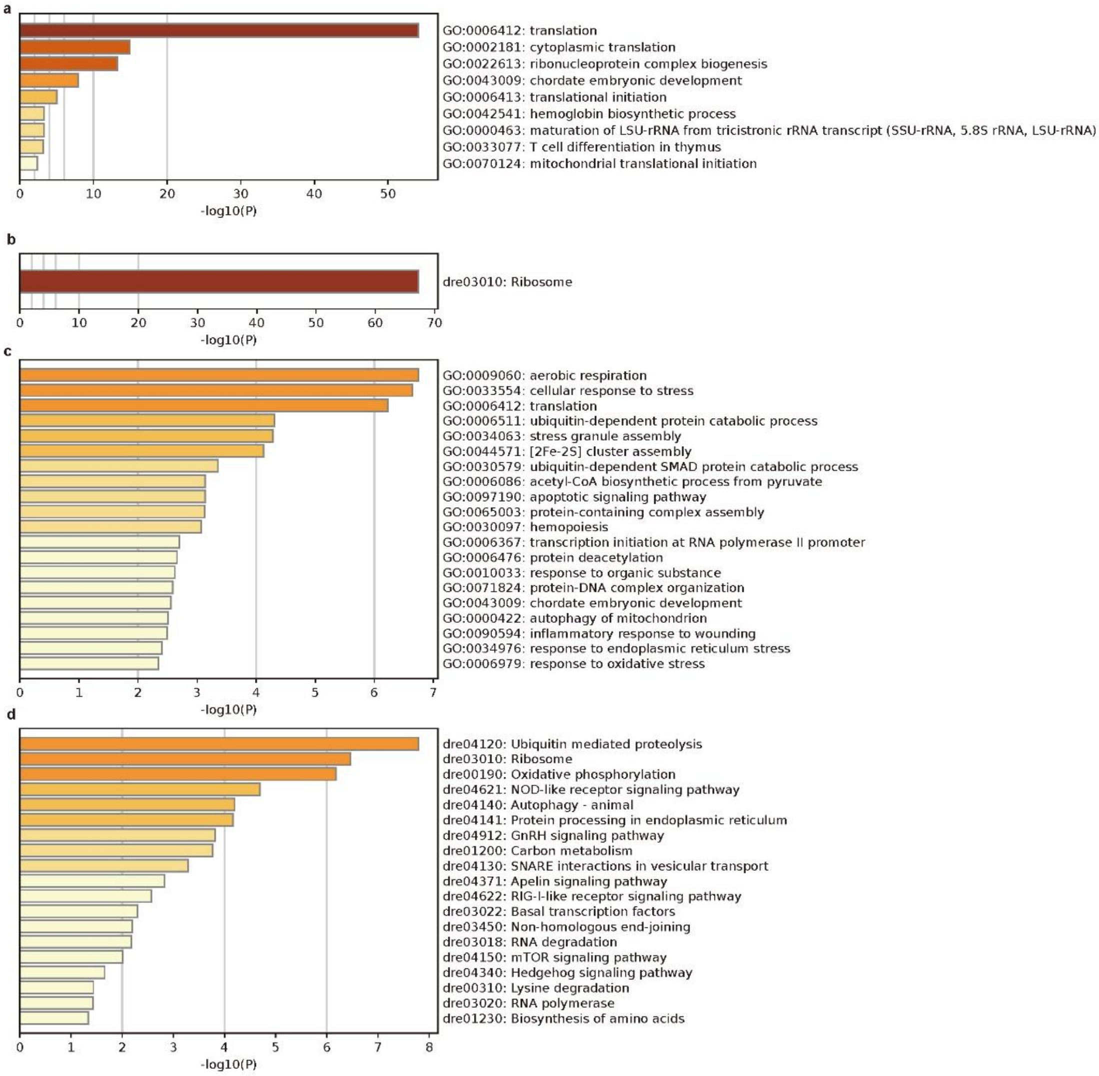

Figure S9. GO enrichment and KEGG enrichment analysis of core genes in the pink and blue modules.

(a) GO enrichment for biological processes of core genes for pink module. (b) KEGG enrichment of core genes for pink module. (c) GO enrichment for biological processes of core genes for blue module. (d) KEGG enrichment of core genes for blue module.

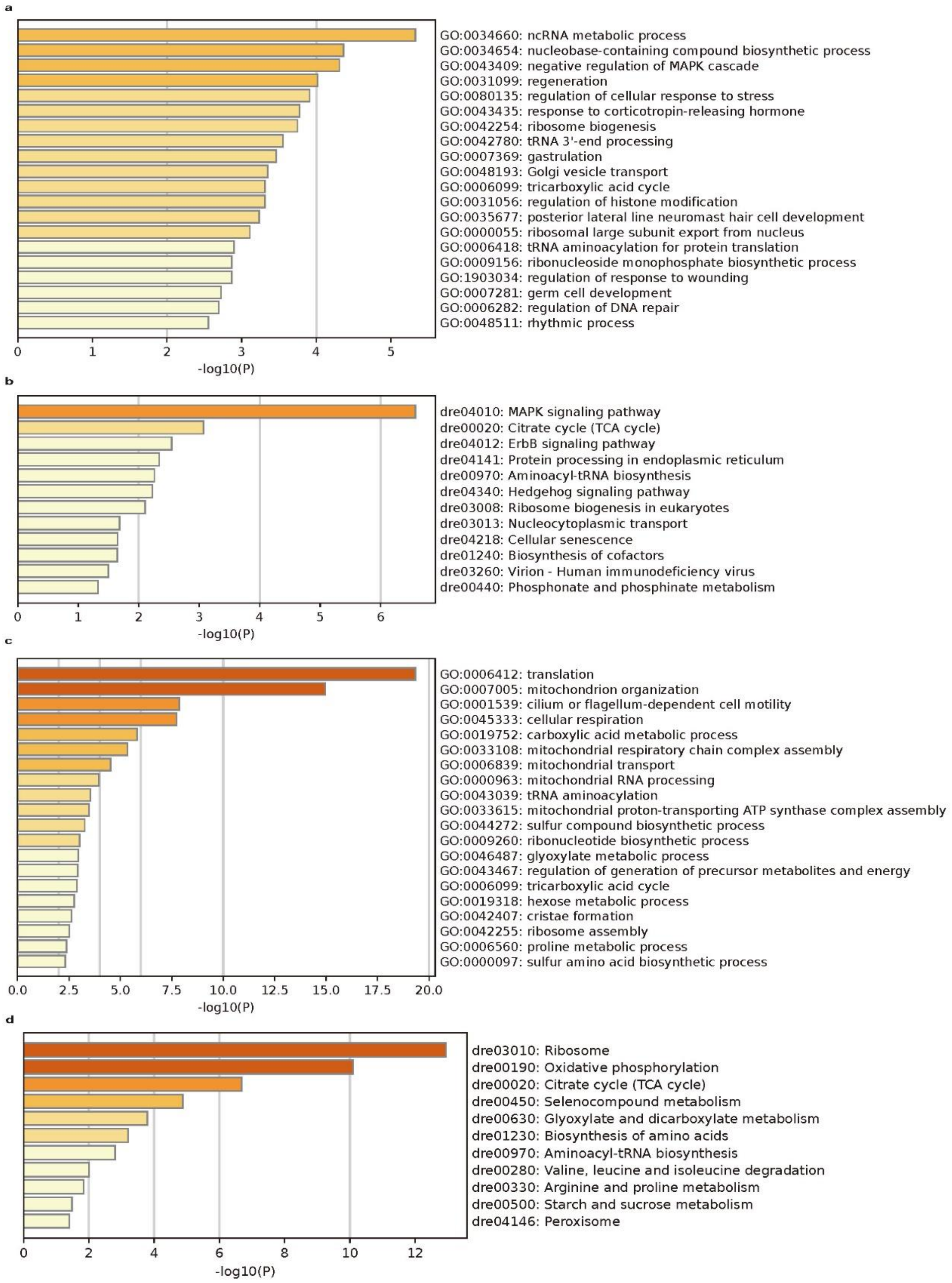

Figure S10. GO enrichment and KEGG enrichment analysis of core genes in the red and green modules.

(a) GO enrichment for biological processes of core genes for red module. (b) KEGG enrichment of core genes for red module. (c) GO enrichment for biological processes of core genes for green module. (d) KEGG enrichment of core genes for green module.

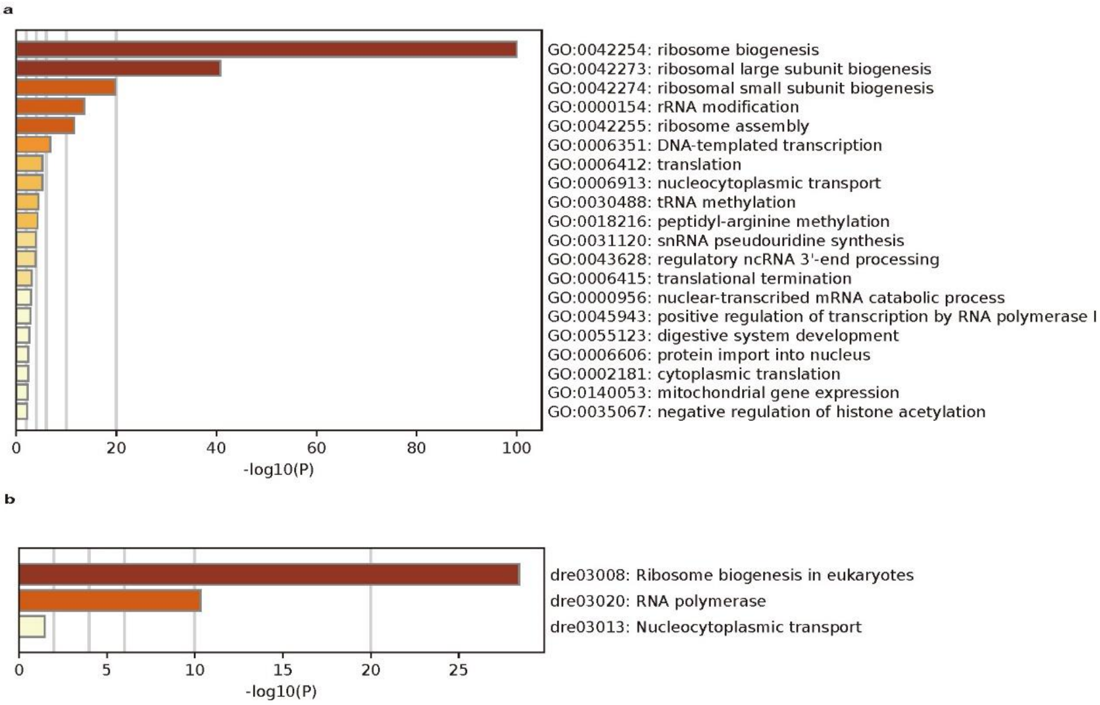

Figure S11. GO enrichment and KEGG enrichment analysis of core genes in the yellow module. (a) GO enrichment for biological processes of core genes for yellow module. (b) KEGG enrichment of core genes for yellow module.

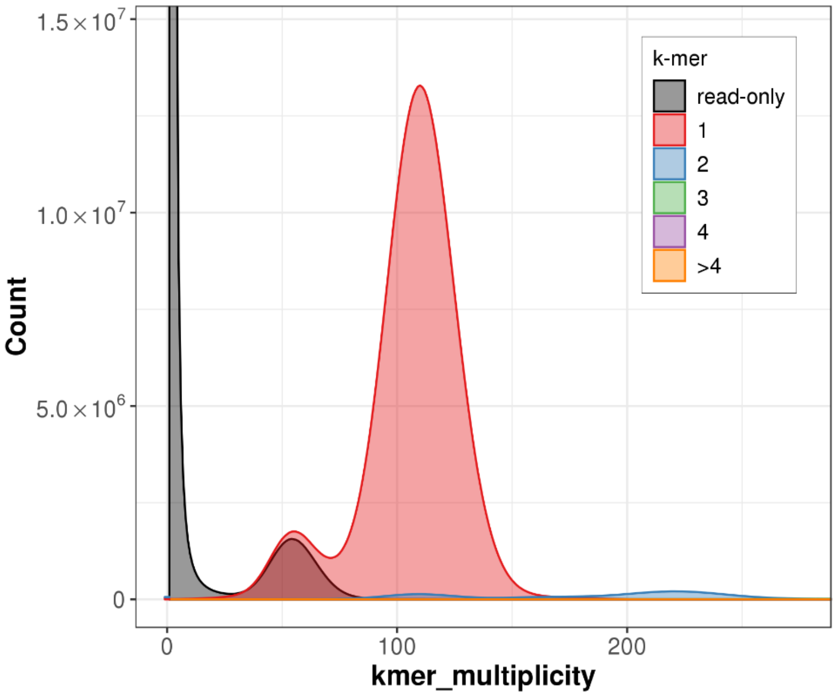

Figure S12. Merqury copy number spectrum plot for assembled genome of *S. ocellatus*.
